# The Doctor’s Burden

**Published:** 1900-11

**Authors:** E. Miller Reid


### The Doctor's Burden.

Some years ago I received a call I will never forget. It was January of much such a winter as we had last year. The night was fearfully dark, moonless and starless, and the fine icy snow falling densely obliterated the street lamps, if, indeed, any remained lighted. There was more than a foot of snow on the ground, and this depth was increasing rapidly; the wind was blowing sixty miles an hour, and piling the snow into almost impassable drifts, and the cold was beyond description. In short, a first-class blizzard had possession of the city and was holding high revel in the streets. I had had a hard day, had just returned from a long trip completely worn out, and at twelve o'clock was just getting warm and comfortable in bed, and devoutly praying that no one would require my services for some hours to come, when my bell began to ring in a way that threatened to snap the wires. The cold struck me as soon as I arose, and with chattering teeth and husky voice I demanded to know who wanted me.

From chattering teeth and husky voice came back, "Mrs. So-and-so wants you at once, her baby is dying." Mrs. So-and-so lived two miles, as the crow flies, and good three miles by the crooked streets through which I had to drive, but the call represented duty, and in those days when duty called I generally responded promptly.

I invited the messenger boy to return with me, but he declined vigorously. He wouldn't go back "for nothing"; he was going to stay at his aunt's, in the next street, he was, and he wouldn't be in my place, that he wouldn't, not if anybody was to offer him five dollars; no, sir.

My coachman had gone home sick, so I had to harness the horses. As I do not want to harrow the felings of my audience I will not tell how long it took me to make those three miles, how often the sleigh upset, how often the horses fell, or how many times they stuck in the drifts, from which it was almost impossible to extricate them. We had to go the whole distance at a snail's pace, and a very slow snail at that. The streets were deserted, I did not see two persons during the entire drive. I was stiff with cold and my breath froze in icicles on my mustache; in short I was freezing.

The mind works queerly at such times. I was always fond of rhyming, and these crazy rhymes began to weave through my brain:

Dark was the night, the snow fell fast,
The bitter winds did blow,
When out into the tempest wild
The doctor had to go.

He was a slender, fragile man,
A man built much like me,
And quite unfit to breast the gale
That drove in from the sea.

But 'twas his sweetheart who was ill,
So he fought on and on,
Till he fell down and lay quite still,
For all his strength was gone.

The gale drove on before it ran,
A lovely white mist wraith,
And ran and ran until it stood
Right in the doctor's path.

"She's dead and is a ghost," he cried
Because I could not treat her.
I'll die and be a spectre too,
And then I'll "spec-ter" meet her.

Then I began to doze, and the next thing I knew the horses had stopped, but this time it was in front of the house to which I had been called. They had been there before and knew the place. The house was dark and there was no sign of anyone being up.

I rang and rang, then knocked and knocked, then rang, then knocked, all the time growing colder and colder in the cutting blasts that swept the street. At last when my patience was completely exhausted a head was thrust from an upstairs window and a shrill feminine voice called: "That you, Doctor? Oh, Doctor, we thought baby had swallowed a pin, but after we had sent the boy we found the pin. You won't charge for this visit, will you?"

Not charge! Great Scott! An Indian war was raging at the time and I was mad enough to have charged single-handed every disaffected red skin in the wild and woolly West up the sheer wall of a cañon ten thousand feet high. Not charge! I charged all the way home; I did not get cold going back; I didn't make any more rhymes; but I did use a good many strong biblical terms, perhaps not wisely, but I know I used them well.

I have always been liberal in my views in regard to religious denominations.

As far as I am concerned a man may be Methodist, Episcopalian, Lutheran, Roman Catholic, Buddhist, Mohammedan, Hard-shell Baptist, or Quaker Oats, but since that night I have specially favored Christian Science; any science, creed, profession, or call it what you will, that will enable a tired physician to lie snug and warm in his bed and give absent treatment on stormy nights commends itself to me.—*E. Miller Reid, M. D., in Medical Gleaner.*

